# Insomnia Is Associated With Frequency of Suicidal Ideation Independent of Depression: A Replication and Extension of Findings From the National Health and Nutrition Examination Survey

**DOI:** 10.3389/fpsyt.2020.561564

**Published:** 2020-09-18

**Authors:** Zach Simmons, Lance D. Erickson, Dawson Hedges, Daniel B. Kay

**Affiliations:** ^1^ Department of Psychology, Brigham Young University, Provo, UT, United States; ^2^ Department of Sociology, Brigham Young University, Provo, UT, United States

**Keywords:** insomnia, suicidal ideation, suicidality, sleep, depression, short sleep

## Abstract

**Objective:**

Insomnia is associated with suicidality, although the mechanisms of this association are unclear. This study sought to replicate previous findings showing that insomnia symptoms but not sleep duration are associated with frequency of suicidal ideation in adults. We further investigated whether depression or sleep duration moderates the association between insomnia symptoms and frequency of suicidal ideation.

**Materials and Methods:**

We used the 2005–2006 cycle of the National Health and Nutrition Examination Survey to replicate previously reported findings from the 2007–2008 cycle. We used ordered logistic regression to determine whether insomnia symptoms were associated with frequency of suicidal ideation independently of depression and other potential confounds. To extend these findings, we tested whether depression or sleep duration moderated the association between insomnia symptoms and frequency of suicidal ideation. We further replicated these findings in parallel analyses using the combined data from the 2005–2006 and 2007–2008 cycles.

**Results:**

This study replicated previous results showing that insomnia symptoms are associated with frequency of suicidal ideation in the NHANES 2005–2006 cycle (OR = 1.09, *p* < 0.05), even after adjusting for potentially confounding variables, including depression. Neither depression nor sleep duration moderated this association. Difficulty with sleep maintenance insomnia symptoms were most robustly associated with frequency of suicidal ideation (OR ≥ 1.97, *p* < 0.05). Sleep duration was not robustly associated with suicidal ideation.

**Conclusions:**

In this study, we found that insomnia symptoms were uniquely associated with frequency of suicidal ideation. This association cannot be explained by the shared association with depression or sleep duration.

## Introduction

Insomnia, including difficulty falling asleep, maintaining sleep, or falling back to sleep, is a common symptom of individuals with suicidality (1, 2). Suicide has become a leading cause of death worldwide (3). In the United States alone, suicide accounted for nearly 45,000 deaths in 2016, a number that has increased by nearly 30 percent since 1999 (4). The rising incidence of suicide emphasizes the need to identify and treat associated factors. Suicidal ideation is a precursor to suicide that has been linked to sleep disturbance (5) and insomnia (6, 7). Although depression is a main target for identifying suicidality, insomnia symptoms might outperform depression in predicting suicidal ideation (8). Further evidence suggests that treatment of insomnia through cognitive-behavioral therapy for insomnia (9) and through controlled-release zolpidem (10) can reduce suicidal ideation. As a potentially modifiable risk factor, insomnia may prove to be a useful target for suicide prevention. The purpose of this study was to replicate and extend findings that suggest that insomnia is associated with frequency of suicidal ideation and to explore the role of depression or sleep duration in this association.

Depression is associated with both insomnia (2, 11) and suicidal ideation (12, 13). While some studies suggest that insomnia is associated with suicidal ideation independent of depression symptoms (7, 14–16), others suggest that depression plays a key role in the association between insomnia and suicidal ideation (17, 18). Replication studies with large samples are needed to better establish the role of depression in the association between insomnia and suicide.

Both short and long sleep duration have been linked to suicidal ideation (19, 20). Findings are mixed, though, on whether depression affects this association. At least one study found that short sleep duration was associated with suicidal ideation independent of depression in adults (21), whereas other studies found that depression better explains the association between sleep duration and suicidality (22, 23). Among individuals who have suicidal ideation, shorter sleep duration, and poorer sleep quality predicted next day increases in suicidal ideation even when adjusting for depression severity. Some researchers have proposed that short or long sleep duration may interact with insomnia, such that the two combined result in greater suicidality (22, 24).

To investigate further the association between insomnia and suicidality, we sought to replicate and extend previous findings from Chakravorty and colleagues. They showed that insomnia symptoms, but not sleep duration, were associated with the frequency of suicidal ideation independent of depression (22). We also sought to further evaluate whether depression or self-reported sleep duration moderates the association between insomnia symptoms and the frequency of suicidal ideation.

## Materials and Methods

### Participants

We used data from the National Health and Nutrition Examination Survey (NHANES) collected by the United States’ Centers for Disease Control and Prevention. The NHANES occurs in multiple biannual cycles. Statistical weighting renders the data representative of the non-institutionalized population of the United States. For this study, we used data from the 2005–2006 cycle to replicate and extend results previously reported from the 2007–2008 cycle (22). We included 4,773 adult participants from the 2005–2006 cycle who had data available in the public-use dataset. In supplemental parallel analyses, we include the respondents from the 2005–2006 cycle and 5,707 respondents from the 2007–2008 cycle.

### Suicidal Ideation

We determined the frequency of suicidal ideation using item 9 on the Patient Health Questionnaire-9 (PHQ-9) (25), which asks how often people have thought it would be better if they were dead or have had thoughts of hurting themselves over the past 2 weeks. Responses included 0, “Not at all”; 1, “Several days”; 2, “More than half the days”; and 3, “Nearly every day”.

### Insomnia Symptom Severity

Three items from the Functional Outcomes of Sleep Questionnaire, a validated self-report assessment of functional impairment due to sleep disturbance (26), assessed insomnia: trouble with initially falling asleep, nighttime awakenings with trouble falling back to sleep, and early-morning awakenings with trouble falling back to sleep. Participants’ responses were based on sleep in the past month and ranged from 0, “NEVER” to 4, “ALMOST ALWAYS”. Similar to how Chakravorty et al. defined global insomnia, we defined global insomnia as the sum of these three items (22).

### Depression

We used the PHQ-9, a validated measure of the Diagnostic and Statistical Manual of Mental Disorders, fourth edition (DSM-IV) criteria for depression (27), to categorize participants as being depressed or not depressed. This nine-item self-report survey assessed participants’ symptoms of depression over the prior 2 weeks. The scoring of these symptoms was 0, “Not at all”; 1, “Several days”; 2, “More than half the days”; and 3, “Nearly every day.” A tenth question assesses the difficulty these symptoms cause in important areas of participants’ life, with a scoring of 0 “Not difficult at all,” 1, “Somewhat difficult”; 2, “Very difficult”; and 3, “Extremely difficult.” The three criteria for depression were (1) experiencing little interest in doing things and/or feeling down, depressed, or hopeless at least more than half the days in the previous 2 weeks, (2) at least five different depressive symptoms in at least half of the days in the past 2 weeks, and (3) that the depression symptoms created at least somewhat difficult problems in major areas of their life. We coded respondents as 1, depressed and 0, not depressed.

### Sleep Duration

As a part of the Functional Outcomes of Sleep Questionnaire, participants were asked “How much sleep do you usually get at night on weekdays or workdays?” in whole hours (26). Participants were categorized by their hours of average sleep duration. Because of small sample sizes in the groups with extreme sleep durations, we combined all participants with ≤4 h into a single group and all participants with ≥10 h into another group.

### Demographic Variables and Other Covariates

The NHANES collected self-reported demographic variables and other covariates. Respondents reported their age in years and their gender as male or female. The NHANES presented race-ethnicity data as five categories: non-Hispanic White, non-Hispanic Black, Mexican American, Other Hispanic, and Other race-ethnicity (including Multi-Racial). For our analysis, we included categories for non-Hispanic White, non-Hispanic Black, Mexican American, and Other, which included the “Other Hispanic” and “Other race” categories. The poverty-to-income ratio (PIR) was a continuous variable defined as the total family income divided by the poverty threshold, as determined by the United States Census Bureau for the year of the interview, and therefore, references a family’s or individual’s financial standing relative to the poverty level. A ratio of 3, for example, means that the income was three times greater than the poverty level for the respondent. Respondents were asked if they had at least 12 alcoholic drinks during the last year. If they answered yes to this question, the follow-up question asked the number of drinks they had during the past week, month, or year (i.e., respondents could report a number in the metric most convenient for them). We recoded these data to represent the number of alcoholic drinks consumed per week. Alcoholic binges per week was the average number of times per week the respondent reported drinking five or more alcoholic drinks in a single day over the past year. Unfortunately, measures that capture participants’ levels of physical activity and exercise were not available in the 2005–2006 cycle as was done in the 2007-2008 cycle. **Table 1** shows the demographic characteristics of the 2005–2006 replication sample. We report the demographic characteristics of the combined 2005–2006 and 2007–2008 sample in **Supplemental Table 1**.

**Table 1:** Demographic characteristics of the replication sample (NHANES 2005–2006 cycle) and frequency of suicidal ideation according to each demographic characteristic.

Characteristic	Overall sample (*N* = 4,773)	Not at all (*n* = 4,578)	Several days (*n* = 133)	More than half the days (*n* = 39)	Nearly every day (*n* = 28)	Percent imputed
Age	48(19)	48	48	45	43	0.00
Gender, female	52%	52%	56%	71%	72%	0.00
Race-ethnicity						0.00
Non-Hispanic white	72%	72%	57%	58%	37%	
Non-Hispanic black	11%	11%	13%	16%	24%	
Mexican American	8%	8%	10%	15%	13%	
Other	9%	8%	19%	11%	26%	
Poverty-to-income	2.66(1.64)	2.71	1.85	1.66	1.37	4.76
Educational attainment						2.77
Less than 9th grade	7%	7%	10%	12%	16%	
9th through 11th	11%	11%	15%	19%	23%	
High school diploma	25%	25%	30%	28%	30%	
Some college	31%	31%	30%	35%	26%	
College graduate	26%	27%	15%	6%	5%	
Marital status						1.63
Married	61%	61%	40%	32%	26%	
Widowed	7%	7%	5%	6%	4%	
Divorced	11%	11%	23%	12%	26%	
Separated	3%	3%	2%	19%	1%	
Never Married	11%	11%	21%	16%	34%	
Cohabiting	8%	8%	9%	16%	10%	
Smoking status						0.08
Non-smoker	51%	51%	52%	57%	42%	
Ex-smoker	25%	25%	17%	8%	16%	
Current smoker	24%	24%	32%	34%	42%	
Alcoholic drinks/week	3.26 (8.43)	3.21	3.38	4.11	9.40	9.03
Alcoholic binges/week	0.14 (.88)	0.13	0.18	0.37	0.73	12.42
Depression	3%	1%	30%	73%	92%	9.47
Overall insomnia	3.67 (3.25)	3.56	5.88	6.57	7.89	—^a^
Difficulty falling asleep						0.00
Never	40%	40%	31%	23%	22%	
Rarely	23%	23%	8%	10%	5%	
Sometimes	22%	22%	26%	16%	18%	
Often	9%	8%	13%	36%	20%	
Always	7%	7%	22%	16%	35%	
Difficulty maintaining sleep						0.10
Never	35%	36%	19%	8%	3%	
Rarely	21%	21%	9%	14%	2%	
Sometimes	24%	24%	30%	21%	20%	
Often	14%	13%	23%	40%	45%	
Always	7%	7%	19%	17%	29%	
Early morning awakenings						0.13
Never	43%	43%	36%	26%	16%	
Rarely	20%	21%	12%	7%	4%	
Sometimes	19%	20%	20%	12%	17%	
Often	11%	11%	13%	34%	30%	
Always	6%	6%	19%	22%	33%	
Sleep duration (h)						0.21
<= 4	5%	4%	14%	23%	26%	
5	9%	8%	16%	20%	20%	
6	23%	23%	20%	16%	25%	
7	30%	31%	15%	11%	8%	
8	27%	27%	22%	22%	7%	
9	5%	5%	5%	6%	14%	
>= 10	3%	2%	7%	2%	1%	

^a^Overall insomnia is a composite of Difficulty falling asleep, Difficulty maintaining sleep, and early morning awakenings. It was created post-imputation for each imputed dataset. N = 4,773. M(SD).

### Statistical Analysis

To replicate analyses from the Chakravorty et al. article, we used the 2005–2006 cycle of the NHANES. We treated missing data using multiple imputation with chained equations (22). Multiple imputation makes a realistic missing-at-random assumption, and the chained-equations approach allowed for imputation conditional on the distribution of the missing data (e.g., nominal, binary, continuous). We created 20 imputed datasets (28) using *mi impute* in Stata 16.1. Datasets were separated by 200 iterations based on graphical diagnostics that indicated that the imputation model converged well before that point (29). We estimated all models on the resulting datasets separately and then combined them using Rubin’s rules with Stata’s *mi estimate* prefix. See the final column in **Table 1** for the percent of data in each variable that were imputed. We used Stata’s *svy* prefix to incorporate the sampling design to adjust the standard errors for the clustered nature of the data and weights to make estimates representative of the noninstitutionalized population of the United States.

We estimated a series of multivariable ordered logistic regression models of frequency of suicidal ideation adjusted for age in years, ethnicity, educational attainment, marital status, poverty-to-income ratio, alcohol use, and smoking. Chakravorty et al. used three nested models: a bivariate model, a model adjusted for a variety of potential confounding variables, and a model that also adjusted for depression (22). To replicate these analyses, we calculated three ordered logistic regression models (**Table 2**). Model 1 included global insomnia, Model 2 included sleep duration, and Model 3 included both global insomnia and sleep duration. Estimates of each of these models were adjusted for depression, demographic variables, and other covariates. Because a measure of global insomnia symptoms that combines individual symptoms may mask the relationship between particular symptoms of suicidal ideation, we also estimated ordered logistic regression models for each component of global insomnia symptoms adjusted for depression and the other covariates not including for sleep duration (**Table 3**). These models treated the items that comprised the global insomnia symptoms measure in their original metrics.

**Table 2:** Insomnia and sleep duration as predictors of suicidal ideation in the replication sample (NHANES 2005–2006 cycle): Odds ratios from ordered logistic regression.

	Model 1		Model 2		Model 3	
	OR	95% CI	OR	95% CI	OR	95% CI
Global insomnia	1.11**	1.04–1.19			1.09*	1.00–1.19
Sleep duration (h)						
<= 4			3.85*	1.42–1.40	2.83*	1.10–7.32
5			2.93*	1.14–7.50	2.41	0.96–6.09
6			1.50	0.71–3.17	1.37	0.65–2.90
7			—		—	
8			1.45	0.56–3.73	1.56	0.60–4.09
9			2.78	0.68–11.33	2.88	0.66–12.51
>= 10			3.14	0.36–27.03	3.33	0.38–29.30
Age	1.01	1.00–1.03	1.01	1.00–1.03	1.01	0.99–1.03
Female	0.91	0.45–1.84	1.03	0.51–2.10	0.94	0.47–1.87
Race-ethnicity						
Non-Hispanic White	—		—		—	
Non-Hispanic Black	1.23	0.69–2.20	1.11	0.62–1.98	1.18	0.67–2.07
Mexican American	1.76	0.89–3.49	1.69	0.89–3.24	1.85	0.96–3.56
Other	3.42*	1.29–9.08	3.70*	1.44–9.49	3.70*	1.44–9.52
Educational attainment						
Less than 9th grade	—		—		—	
9th through 11th	1.06	0.40–2.78	1.09	0.45–2.67	1.06	0.43–2.62
High school diploma	0.91	0.41–2.00	0.91	0.41–2.04	0.90	0.40–2.03
Some college	0.98	0.42–2.28	0.97	0.42–2.20	0.97	0.43–2.22
College graduate	0.72	0.25–2.11	0.76	0.26–2.24	0.74	0.25–2.21
Poverty-to-income	0.79*	0.67–0.95	0.80*	0.66–0.97	0.81*	0.67–0.98
Marital status						
Married	—		—		—	
Widowed	0.87	0.25–2.97	0.83	0.23–3.03	0.83	0.22–3.12
Divorced	1.60	0.81–3.15	1.61	0.82–3.15	1.63	0.84–3.19
Separated	0.87	0.32–2.32	0.82	0.31–2.19	0.81	0.30–2.21
Never married	2.01	0.96–4.20	1.92	0.92–4.00	1.94	0.93–4.03
Cohabiting	1.48	0.55–3.97	1.38	0.49–3.83	1.43	0.53–3.83
Smoking status						
Non-smoker	—		—		—	
Ex-smoker	.50	0.24–1.03	0.56	0.28–1.12	0.53	0.26–1.08
Current smoker	.70	0.40–1.23	0.75	0.44–1.27	0.72	0.42–1.23
Alcoholic drinks/week	1.01	0.98–1.04	1.01	0.99–1.04	1.01	0.98–1.03
Alcoholic binges/week	1.07	0.85–1.35	1.07	0.84–1.35	1.08	0.86–1.36
Depression	55.49***	21.82–141.11	63.35***	27.72–144.79	52.57***	22.05–125.32

N = 4,773. *p < 0.05, **p < 0.01, ***p < 0.001.

**Table 3:** Components of insomnia as predictors of reported frequency of suicidal ideation in the replication sample (NHANES 2005–2006 cycle): odds ratios from ordered logistic regression.

	Model 1		Model 2		Model 3	
	OR	95% CI	OR	95% CI	OR	95% CI
Difficulty falling asleep						
Never	—					
Rarely	0.45*	0.22–0.88				
Sometimes	1.07	0.55–2.08				
Often	1.50	0.80–2.81				
Always	1.34	0.68–2.61				
Difficulty maintaining sleep						
Never			—			
Rarely			0.92	0.42–2.02		
Sometimes			1.97*	1.05–3.72		
Often			3.62**	1.72–7.64		
Always			3.53**	1.56–7.96		
Early morning awakenings						
Never					—	
Rarely					0.64	0.32–1.26
Sometimes					1.04	0.64–1.70
Often					1.52	0.88–2.64
Always					2.22	0.84–5.87
Age	1.01	1.00–1.03	1.01	1.00–1.03	1.01	1.00–1.03
Female	0.97	0.47–2.01	0.87	0.43–1.77	0.98	0.47–2.05
Race-ethnicity						
Non-Hispanic White	—		—		—	
Non-Hispanic Black	1.19	0.66–2.11	1.24	0.69–2.23	1.21	0.68–2.15
Mexican American	1.66	0.84–3.28	1.79	0.90–3.59	1.67	0.85–3.29
Other	3.35*	1.26–8.90	3.40*	1.28–9.02	3.47*	1.29–9.30
Educational attainment						
Less than 9th grade	—		—		—	
9th through 11th	1.11	0.43–2.90	1.01	0.38–2.64	1.08	0.41–2.83
High school diploma	0.94	0.43–2.07	0.86	0.38–1.91	0.96	0.44–2.07
Some college	1.03	0.45–2.38	0.96	0.41–2.23	1.02	0.44–2.35
College graduate	0.77	0.26–2.25	0.70	0.23–2.16	0.77	0.26–2.30
Poverty-to-income	0.80*	0.67–0.96	0.80*	0.67–.95	0.80*	0.67–0.96
Marital status						
Married	—		—		—	
Widowed	0.87	0.26–2.86	0.86	0.25–3.01	0.86	0.25–2.96
Divorced	1.58	0.82–3.05	1.61	0.83–3.11	1.56	0.80–3.03
Separated	0.83	0.32–2.17	0.85	0.28–2.53	0.87	0.35–2.15
Never Married	2.04	0.98–4.25	2.07	0.96–4.47	1.95	0.91–4.18
Cohabiting	1.47	0.53–4.12	1.51	0.58–3.94	1.46	0.53–4.04
Smoking status						
Non-smoker	—		—		—	
Ex-smoker	0.51	0.25–1.03	0.47	0.22–1.01	0.52	0.25–1.09
Current smoker	0.74	0.42–1.30	0.74	0.42–1.29	0.71	0.41–1.22
Alcoholic drinks/week	1.01	0.98–1.04	1.01	0.98–1.03	1.01	0.98–1.04
Alcoholic binges/week	1.05	0.83–1.33	1.07	0.86–1.35	1.06	0.84–1.33
Depression	66.18***	26.68–164.17	55.07***	22.16–136.85	64.29***	25.42–162.60

N = 4,773. *p < 0.05, **p < 0.01, ***p < 0.001.

To extend previous findings, we also evaluated whether depression or sleep duration moderated the association between global insomnia symptom severity and frequency of suicidal ideation (**Table 4**). To do this, we estimated two separate models. The first model adjusted for covariates and included a global insomnia and depression interaction term. The second model also adjusted for covariates and included a global insomnia by sleep duration interaction term. In the regression models, statistically significant interactions between insomnia symptom severity and sleep duration or insomnia symptom severity and depression represented evidence that depression or sleep duration, respectively, moderated the insomnia-suicidal ideation relationship.

**Table 4:** Moderation analyses showing that neither sleep duration nor depression significantly moderated the association between insomnia and frequency of suicidal ideation in the replication sample (NHANES 2005–2006 cycle).

	OR	95% CI	*p*-value
Model 1			
Insomnia	1.15	0.93–1.41	0.183
Sleep duration (h)			
<=4	8.22**	2.51–26.92	0.002
5	2.16	0.63–7.38	0.195
6	1.34	0.35–5.16	0.647
7	—		
8	2.17	0.74–6.39	0.141
9	6.39**	1.87–21.79	0.007
>=10	1.54	0.13–18.53	0.709
Sleep duration X insomnia			
<=4	0.86	0.66–1.12	0.237
5	1.00	0.77–1.30	0.979
6	0.99	0.75–1.31	0.950
7	—		
8	0.92	0.71–1.20	0.517
9	0.82	0.62–1.09	0.150
>=10	1.18	0.81–1.72	0.364
**Model 2**			
Insomnia	1.11*	1.03–1.20	0.013
Depression	87.52***	17.63–434.39	<0.001
Depression X insomnia	0.93	0.77–1.12	0.397

Each model includes adjustments for age, gender, race, education, poverty-to-income ratio, marital status, smoking status, alcohol consumption, and binge drinking. Model 1 is adjusted for depression and Model 2 is adjusted for sleep duration. N = 4,773. *p < 0.05, **p < 0.01, ***p < 0.001.

All aforementioned analyses were further replicated in the combined 2005–2006 and 2007–2008 sample. We report the statistical results of the analyses in the combined sample in the **Supplementary Material**.

## Results

**Table 2** reports the three separate models with odds ratios for self-reported frequency of suicidal ideation of the imputed sample from the NHANES 2005–2006 data sets. In Model 1, global insomnia, Other race-ethnicity, lower poverty-to-income ratio, and greater depression were significantly associated with higher frequency of suicidal ideation. In Model 2, sleep duration (≤4 and 5 h) was additionally associated with greater suicidal ideation. In Model 3, the full model, higher global insomnia, sleep duration of less than or equal to 4 h, Other race-ethnicity, lower poverty-to-income ratio, and greater depression were associated with higher frequency of suicidal ideation. These analyses were repeated in the combined NHANES 2005–2006 and 2007–2008 sample (**Supplemental Table 2**). In the combined sample, greater global insomnia, younger age, Other race-ethnicity, lower poverty-to-income ratio, having never been married, and greater depression were associated with higher frequency of suicidal ideation. Being an ex-smoker was associated with less frequent suicidal ideation.

**Table 3** reports three models, one for each symptom of insomnia – difficulty falling asleep, difficulty maintaining sleep, and early-morning awakening. These models used the NHANES 2005–2006 dataset and adjusted for demographic variables and other covariates. Sleep duration was not included in these models to match the original study that these replicate (22). In these models, “Rarely” having difficulty falling asleep was associated with lower frequency of suicidal ideation. “Sometimes,” “Often,” and “Always” having difficulty maintaining sleep were associated with greater frequency of suicidal ideation. There were no associations between early-morning awakenings and frequency of suicidal ideation in the replication sample. **Supplementary Table 3** reports three parallel models using the NHANES combined 2005–2006 and 2007–2008 sample. In this combined sample, “Often” and “Always” having difficulty falling asleep were associated with higher frequency of suicidal ideation. “Sometimes,” “Often,” and “Always” having difficulty maintaining sleep were associated with higher frequency of suicidal ideation. Finally, “Often” and “Always” having early-morning awakenings were associated with higher frequency of suicidal ideation.

**Table 4** shows the results for the moderation analyses. We constructed two models, one to test for a global insomnia by depression interaction and the other to test for a global insomnia by sleep duration interaction. These moderation analyses failed to find a significant interaction for depression or sleep duration in predicting frequency of suicidal ideation. In the combined sample, that used the 2005–2006 and 2007–2008 dataset, these interactions approached significance at the trend level (**Supplemental Table 4**).

## Discussion

Using the 2005–2006 NHANES cycle as a replication sample and the larger combined 2005–2006 and 2007–2008 sample that included 10,480 participants, we replicated previous findings reported from the 2007–2008 NHANES cycle that showed an association between worse insomnia symptoms and a higher frequency of suicidal ideation (22). This association remained even after accounting for potential confounds including age, ethnicity, educational attainment, marital status, poverty-to-income ratio, alcohol use, smoking, depression, and sleep duration. Analyses of each component symptom of insomnia in our combined sample suggested that the three hallmark symptoms of insomnia are individually associated with an increase in suicidal ideation. In the smaller replication (2005–2006) and the originally reported (2007–2008) samples, maintenance insomnia symptoms were more robustly associated with suicidal ideation than onset or early morning problems. Studies on which symptom of insomnia is most strongly related to suicidal ideation are sparse. One study found that maintenance insomnia symptoms were more strongly associated with suicidal ideation (30). Previous findings using research evidence from military veterans suggests that insomnia symptoms are only indirectly related to suicidal ideation *via* psychopathology, such as depression and alcohol use (31–33). This conclusion conflicts with another study that found sleep disturbance outperformed depression as a predictor of suicidal ideation in young adults in the military (8). The present results suggest that insomnia is a direct risk factor for suicidal ideation independent of psychopathology (i.e., depression and alcohol use) in a nationally representative sample.

As expected, we found that depression was the strongest predictor of suicidal ideation as shown in **Tables 2**–**4** and **Supplemental Tables 2–4**. One study suggested that patients with insomnia in conjunction with depression may experience greater suicidal ideation than those with depression alone (34). In the present study, insomnia and depression did not significantly moderate their individual relationships with suicidal ideation. Previous studies have found an association between sleep duration and suicidality (19, 22, 23). Some authors have suggested that insomnia with short sleep duration is a more severe form of insomnia (24, 35). Insomnia with short sleep has been linked to greater rates of mortality (36, 37), suggesting that sleep duration and insomnia interact. In our fully adjusted model from the combined NHANES dataset, we failed to find support for the hypothesis that insomnia symptoms with altered sleep duration represented a greater risk factor for frequency of suicidal ideation. Sleep duration did not significantly moderate the association between global insomnia symptoms and frequency of suicidal ideation. In our model unadjusted for insomnia symptoms, sleep duration was associated with suicidal ideation. This association was not robust when accounting for insomnia and other potential confounds. Our analyses also suggested that younger age, Other race-ethnicity, lower poverty-to-income ratio, and having never married. Being an ex-smoker is a potential protective factor for suicidal ideation. These findings, aside from Other ethnicity (38), are consistent with previous findings (39–42).

Several theories have been proposed to explain the association between sleep disturbance and suicidality. These theories contend that sleep disturbance leads to emotion dysregulation (43), thwarted belongingness (44), disgust with the world (45), and impulsivity (46) thereby increasing suicidality. Due to the secondary nature of the present study, we were not able to test these potential models. Nevertheless, our results help focus the discussion on insomnia as a specific type of sleep disturbance linked to suicidality. We have proposed that insomnia may involve regionalized sleep disturbance in brain networks involved in executive control, self-referential thinking, and affect. Indeed, our previous work suggests that patients with insomnia have less nonrapid eye movement (NREM) sleep-wake differences in the left parieto-frontal cortex, posterior cingula, and lingual areas that have been liked to these processes (47). We have posited that “local sleep deprivation” in these brain regions may predispose individuals to specific daytime impairments (47, 48). Future research is needed to test whether the brain alterations during NREM sleep associated with insomnia lead to specific daytime impairments that thereby put individuals with insomnia at greater risk for suicidality.

This study has several strengths, such as the use of a large dataset weighted to be representative of the non-institutionalized population of the United States, inclusion of several measures of sleep disturbance, and inclusion of several covariates that possibly could confound an association between insomnia and suicidal ideation. In addition, we investigated whether depression or sleep duration might moderate the association we found between insomnia symptoms and suicidal ideation and found that sleep duration and depression did not moderate the association between insomnia symptoms and suicidal ideation. Despite these strengths, however, several factors require consideration when interpreting these findings. The cross-sectional study design we used precludes determining whether insomnia symptoms lead to suicidal ideation or whether suicidal ideation lead to insomnia. While we think it is more likely that insomnia leads to suicidal ideation than that suicidal ideation leads to insomnia, data from longitudinal designs would be critical in better understanding the causal relationship between insomnia and suicidal ideation. A previous study suggested that short sleep duration and sleep disturbance predict higher next-day suicidal ideation (49). Regardless, the association we found between insomnia and suicidal ideation suggests that the role of insomnia in suicidal ideation merits additional research, particularly given the immense personal and public health problems associated with suicidal ideation and that insomnia is potentially preventable and treatable. An additional limitation is that the data we used for our primary independent and dependent variables were based on self-report. Polysomnographic measures of sleep duration might provide a clearer picture of how sleep is associated with suicidality. Similarly, while the questionnaire assessing frequency of suicidal ideation may give some indication of suicidality severity, it does not provide information concerning previous suicide plans or suicidal behavior. Also, due to the nature of the survey, we are blind to those who may have attempted or committed suicide. Categorizing participants into groups of no suicidality, suicidal ideation, suicide attempt, and suicide would help in understanding factors that contribute to the risk of varying degrees of suicidality. Lastly, the public-use version of NHANES does not provide details on race-ethnicity that would allow us to examine the “Other” racial groups separately.

Insomnia symptoms are associated with suicidal ideation independently of depression in models controlling for several potential confounding variables. Further, neither depression nor sleep duration significantly moderated the association between insomnia symptoms and frequency of suicidal ideation. Insomnia is modifiable and as a risk factor for suicidal ideation deserves additional research to establish the mechanism of this association.

## Data Availability Statement

Publicly available datasets were analyzed in this study. This data can be found here: https://wwwn.cdc.gov/nchs/nhanes/ContinuousNhanes/Default.aspx?BeginYear=2005 https://wwwn.cdc.gov/nchs/nhanes/ContinuousNhanes/Default.aspx?BeginYear=2007.

## Author Contributions

ZS and DK conceptualized the project, and LE and DH assisted with the methodology and formal analyses of the project. ZS was predominantly responsible for writing the original draft and all other authors were involved in reviewing and editing the article. DK was the senior author on this project.

## Conflict of Interest

The authors declare that the research was conducted in the absence of any commercial or financial relationships that could be construed as a potential conflict of interest.
