# Supplementary material for: Insomnia Is Associated With Frequency of Suicidal Ideation Independent of Depression: A Replication and Extension of Findings From the National Health and Nutrition Examination Survey
Source: Front Psychiatry. 2020 Sep 18;11:561564. doi: 10.3389/fpsyt.2020.561564 (PMC7530944; doi:10.3389/fpsyt.2020.561564)
Supplement: Supplementary file 1 [file DataSheet_1.docx]

| **Supplemental Table 1.** Demographic characteristics of the combined sample (NHANES 2005-2006 & 2007-2008 cycles) and frequency of suicidal ideation according to each demographic characteristic. | | | | | | |
| --- | --- | --- | --- | --- | --- | --- |
| Characteristic | Overall Sample  (*n* = 10,480) | Not at all  (*n* = 9,989) | Several days  (*n* = 322) | More than  half the days  (*n* = 82) | Nearly every day  (*n* = 87) | Percent imputed |
| Age, years | 50(18) | 50 | 50 | 46 | 49 | 0.00 |
| Gender, female | 52% | 52% | 54% | 67% | 66% | 0.00 |
| Race-ethnicity |  |  |  |  |  | 0.00 |
| Non-Hispanic White | 71% | 71% | 59% | 55% | 47% |  |
| Non-Hispanic Black | 11% | 11% | 12% | 16% | 19% |  |
| Mexican American | 8% | 8% | 11% | 16% | 10% |  |
| Other | 10% | 10% | 18% | 12% | 24% |  |
| Poverty-to-income | 2.57(1.67) | 2.62 | 1.77 | 1.50 | 1.23 | 7.22 |
| Educational attainment |  |  |  |  |  | 2.45 |
| Less than 9th grade | 7% | 7% | 11% | 15% | 18% |  |
| 9th through 11th | 12% | 12% | 17% | 23% | 23% |  |
| High school diploma | 25% | 25% | 28% | 30% | 26% |  |
| Some college | 29% | 29% | 30% | 27% | 27% |  |
| College graduate | 26% | 27% | 14% | 5% | 6% |  |
| Marital status |  |  |  |  |  | 1.76 |
| Married | 61% | 62% | 41% | 36% | 34% |  |
| Widowed | 7% | 6% | 7% | 9% | 5% |  |
| Divorced | 11% | 11% | 18% | 12% | 20% |  |
| Separated | 3% | 3% | 4% | 13% | 9% |  |
| Never Married | 12% | 12% | 22% | 15% | 26% |  |
| Cohabiting | 7% | 7% | 9% | 14% | 7% |  |
| Smoking status |  |  |  |  |  | 0.10 |
| Non-smoker | 52% | 52% | 51% | 46% | 38% |  |
| Ex-smoker | 24% | 25% | 16% | 8% | 17% |  |
| Current smoker | 23% | 23% | 33% | 46% | 45% |  |
| Alcoholic drinks/week | 3.15(8.50) | 3.13 | 3.40 | 3.55 | 5.02 | 8.89 |
| Alcoholic binges/week | 0.13(0.84) | 0.12 | 0.22 | 0.34 | 0.43 | 12.24 |
| Depression | 3% | 2% | 29% | 64% | 87% | 9.26 |
| Global insomnia | 3.70(3.31) | 3.57 | 5.98 | 6.21 | 7.67 | ---^a^ |
| Difficulty falling asleep |  |  |  |  |  | 0.04 |
| Never | 39% | 40% | 26% | 22% | 17% |  |
| Rarely | 22% | 22% | 12% | 14% | 9% |  |
| Sometimes | 22% | 22% | 24% | 18% | 18% |  |
| Often | 10% | 10% | 17% | 24% | 24% |  |
| Always | 8% | 7% | 22% | 21% | 32% |  |
| Difficulty maintaining sleep |  |  |  |  |  | 0.09 |
| Never | 35% | 36% | 19% | 16% | 10% |  |
| Rarely | 20% | 21% | 9% | 12% | 8% |  |
| Sometimes | 24% | 24% | 32% | 19% | 19% |  |
| Often | 13% | 13% | 22% | 33% | 35% |  |
| Always | 8% | 7% | 19% | 19% | 28% |  |
| Early morning awakenings |  |  |  |  |  | 0.11 |
| Never | 44% | 44% | 32% | 31% | 20% |  |
| Rarely | 20% | 20% | 12% | 9% | 9% |  |
| Sometimes | 20% | 20% | 22% | 13% | 18% |  |
| Often | 11% | 10% | 17% | 27% | 24% |  |
| Always | 6% | 6% | 17% | 19% | 30% |  |
| Sleep duration (hours) |  |  |  |  |  | 0.15 |
| <= 4 | 5% | 5% | 14% | 21% | 23% |  |
| 5 | 9% | 9% | 13% | 16% | 16% |  |
| 6 | 23% | 23% | 21% | 20% | 21% |  |
| 7 | 30% | 30% | 18% | 13% | 11% |  |
| 8 | 26% | 26% | 23% | 23% | 17% |  |
| 9 | 5% | 5% | 4% | 5% | 6% |  |
| >= 10 | 2% | 2% | 6% | 2% | 5% |  |

Note: ^a^ Overall insomnia is a composite of difficulty falling asleep, difficulty maintaining sleep, and early morning awakenings. It was created post-imputation for each imputed dataset. *N* = 10,480. *M(SD*).

| **Supplemental Table 2**. Insomnia and sleep duration as predictors of suicidal ideation in the combined sample (NHANES 2005-2006 & 2007-2008 cycles): Odds ratios from ordered logistic regression. | | | | | | | | |
| --- | --- | --- | --- | --- | --- | --- | --- | --- |
|  | Model 1 | |  | Model 2 | |  | Model 3 | |
|  | *OR* | 95*% CI* |  | *OR* | 95*% CI* |  | *OR* | 95*% CI* |
| Global insomnia | 1.11*** | 1.06, 1.15 |  |  |  |  | 1.11*** | 1.06, 1.16 |
| Sleep duration (hours) |  |  |  |  |  |  |  |  |
| <= 4 |  |  |  | 2.19* | 1.20, 4.01 |  | 1.46 | 0.80, 2.67 |
| 5 |  |  |  | 1.60 | 0.85, 2.98 |  | 1.25 | 0.65, 2.39 |
| 6 |  |  |  | 1.25 | 0.86, 1.81 |  | 1.12 | 0.78, 1.62 |
| 7 |  |  |  |  |  |  |  |  |
| 8 |  |  |  | 1.33 | 0.82, 2.17 |  | 1.41 | 0.86, 2.30 |
| 9 |  |  |  | 1.72 | 0.71, 4.16 |  | 1.78 | 0.72, 4.41 |
| >= 10 |  |  |  | 1.91 | 0.63, 5.76 |  | 2.07 | 0.68, 6.33 |
| Age | 1.01* | 1.00, 1.02 |  | 1.01* | 1.00, 1.02 |  | 1.01* | 1.00, 1.02 |
| Female | 0.79 | 0.53, 1.18 |  | 0.87 | 0.59, 1.30 |  | 0.79 | 0.53, 1.17 |
| Race-ethnicity |  |  |  |  |  |  |  |  |
| Non-Hispanic White |  |  |  |  |  |  |  |  |
| Non-Hispanic Black | 0.95 | 0.64, 1.41 |  | 0.89 | 0.62, 1.28 |  | 0.95 | 0.65, 1.38 |
| Mexican American | 1.47 | 0.93, 2.32 |  | 1.37 | 0.87, 2.15 |  | 1.50 | 0.95, 2.38 |
| Other | 2.18* | 1.22, 3.90 |  | 2.19** | 1.25, 3.84 |  | 2.23** | 1.25, 3.96 |
| Educational attainment |  |  |  |  |  |  |  |  |
| Less than 9th grade |  |  |  |  |  |  |  |  |
| 9th through 11th | 0.92 | 0.58, 1.47 |  | 0.96 | 0.61, 1.51 |  | 0.95 | 0.61, 1.48 |
| High school diploma | 0.71 | 0.48, 1.06 |  | 0.72 | 0.47, 1.09 |  | 0.72 | 0.48, 1.08 |
| Some college | 0.87 | 0.53, 1.42 |  | 0.89 | 0.54, 1.47 |  | 0.89 | 0.54, 1.46 |
| College graduate | 0.60 | 0.32, 1.14 |  | 0.62 | 0.33, 1.16 |  | 0.62 | 0.33, 1.17 |
| Poverty-to-income | 0.76*** | 0.66, 0.87 |  | 0.77*** | 0.67, 0.88 |  | 0.77*** | 0.67, .89 |
| Marital status |  |  |  |  |  |  |  |  |
| Married |  |  |  |  |  |  |  |  |
| Widowed | 1.15 | 0.59, 2.23 |  | 1.10 | 0.56, 2.17 |  | 1.11 | 0.56, 2.22 |
| Divorced | 1.35 | 0.83, 2.19 |  | 1.35 | 0.85, 2.16 |  | 1.33 | 0.83, 2.15 |
| Separated | 1.31 | 0.72, 2.39 |  | 1.30 | 0.73, 2.32 |  | 1.29 | 0.71, 2.35 |
| Never Married | 2.10*** | 1.43, 3.07 |  | 2.02** | 1.37, 2.99 |  | 2.03** | 1.37, 3.02 |
| Cohabiting | 1.55 | 0.87, 2.76 |  | 1.49 | 0.84, 2.65 |  | 1.54 | 0.87, 2.73 |
| Smoking status |  |  |  |  |  |  |  |  |
| Non-smoker |  |  |  |  |  |  |  |  |
| Ex-smoker | 0.55** | 0.38, 0.80 |  | 0.59** | 0.41, 0.85 |  | 0.56** | 0.38, 0.81 |
| Current smoker | 0.93 | 0.69, 1.26 |  | 0.98 | 0.72, 1.34 |  | 0.93 | 0.69, 1.27 |
| Alcoholic drinks/week | 1.00 | 0.98, 1.02 |  | 1.00 | 0.98, 1.02 |  | 1.00 | 0.98, 1.02 |
| Alcoholic binges/week | 1.09 | 0.92, 1.29 |  | 1.10 | 0.92, 1.30 |  | 1.10 | 0.93, 1.30 |
| Depression | 36.77*** | 21.00, 64.38 |  | 44.28*** | 26.21, 74.81 |  | 35.64*** | 2.44, 62.14 |
| Note: *N* = 10,480. **p* < 0.05, ***p* < 0.01, ****p* < 0.001. | | | | | | | | |

| **Supplemental Table 3**. Components of insomnia as predictors of reported frequency of suicidal ideation in the combined sample (NHANES 2005-2006 & 2007-2008 cycles): odds ratios from ordered logistic regression. | | | | | | | | |
| --- | --- | --- | --- | --- | --- | --- | --- | --- |
|  | Model 1 | |  | Model 2 | |  | Model 3 | |
|  | *OR* | 95*% CI* |  | *OR* | 95*% CI* |  | *OR* | 95*% CI* |
| Difficulty falling asleep |  |  |  |  |  |  |  |  |
| Never | --- |  |  |  |  |  |  |  |
| Rarely | 0.93 | 0.58, 1.49 |  |  |  |  |  |  |
| Sometimes | 1.41 | 0.95, 2.11 |  |  |  |  |  |  |
| Often | 1.71** | 1.18, 2.48 |  |  |  |  |  |  |
| Always | 1.94** | 1.24, 3.03 |  |  |  |  |  |  |
| Difficulty maintaining sleep |  |  |  |  |  |  |  |  |
| Never |  |  |  | --- |  |  |  |  |
| Rarely |  |  |  | 1.08 | 0.62, 1.89 |  |  |  |
| Sometimes |  |  |  | 2.23*** | 1.52, 3.27 |  |  |  |
| Often |  |  |  | 3.09*** | 2.03, 4.69 |  |  |  |
| Always |  |  |  | 2.59*** | 1.60, 4.19 |  |  |  |
| Early morning awakenings |  |  |  |  |  |  |  |  |
| Never |  |  |  |  |  |  | --- |  |
| Rarely |  |  |  |  |  |  | 0.93 | 0.63, 1.37 |
| Sometimes |  |  |  |  |  |  | 1.25 | 0.91, 1.72 |
| Often |  |  |  |  |  |  | 1.81** | 1.21, 2.71 |
| Always |  |  |  |  |  |  | 1.92* | 1.11, 3.32 |
| Age | 1.01* | 1.00, 1.02 |  | 1.01* | 1.00, 1.02 |  | 1.01* | 1.00, 1.02 |
| Female | 0.81 | 0.54, 1.23 |  | 0.76 | 0.51, 1.15 |  | 0.83 | 0.55, 1.26 |
| Race-ethnicity |  |  |  |  |  |  |  |  |
| Non-Hispanic white | --- |  |  | --- |  |  | --- |  |
| Non-Hispanic black | 0.96 | 0.66, 1.40 |  | 0.95 | 0.64, 1.42 |  | 0.93 | 0.63, 1.37 |
| Mexican American | 1.42 | 0.90, 2.23 |  | 1.48 | 0.92, 2.36 |  | 1.38 | 0.88, 2.18 |
| Other | 2.16* | 1.22, 3.84 |  | 2.19* | 1.21, 3.95 |  | 2.17* | 1.21, 3.88 |
| Educational attainment |  |  |  |  |  |  |  |  |
| Less than 9th grade | --- |  |  | --- |  |  | --- |  |
| 9th through 11th | 0.94 | 0.59, 1.49 |  | 0.90 | 0.57, 1.44 |  | 0.94 | 0.59, 1.50 |
| High school diploma | 0.73 | 0.49, 1.08 |  | 0.68 | 0.46, 1.01 |  | 0.74 | 0.50, 1.09 |
| Some college | 0.88 | 0.54, 1.43 |  | 0.86 | 0.53, 1.39 |  | 0.88 | 0.53, 1.46 |
| College graduate | 0.60 | 0.32, 1.14 |  | 0.59 | 0.30, 1.14 |  | 0.62 | 0.33, 1.18 |
| Poverty-to-income | 0.76*** | 0.66, 0.88 |  | 0.75*** | 0.65, .87 |  | 0.76*** | 0.66, .87 |
| Marital status |  |  |  |  |  |  |  |  |
| Married | --- |  |  | --- |  |  | --- |  |
| Widowed | 1.13 | 0.58, 2.17 |  | 1.18 | 0.61, 2.28 |  | 1.14 | 0.59, 2.21 |
| Divorced | 1.37 | 0.85, 2.20 |  | 1.36 | 0.85, 2.16 |  | 1.34 | 0.83, 2.17 |
| Separated | 1.31 | 0.73, 2.36 |  | 1.32 | 0.70, 2.47 |  | 1.31 | 0.72, 2.37 |
| Never Married | 2.08*** | 1.41, 3.05 |  | 2.13*** | 1.44, 3.14 |  | 2.10*** | 1.43, 3.08 |
| Cohabiting | 1.53 | 0.85, 2.76 |  | 1.63 | 0.94, 2.84 |  | 1.55 | 0.87, 2.77 |
| Smoking status |  |  |  |  |  |  |  |  |
| Non-smoker | --- |  |  | --- |  |  | --- |  |
| Ex-smoker | 0.56** | 0.38, 0.81 |  | 0.55** | 0.38, 0.80 |  | 0.57** | 0.39, 0.83 |
| Current smoker | 0.96 | 0.70, 1.31 |  | 0.96 | 0.71, 1.30 |  | 0.96 | 0.70, 1.30 |
| Alcoholic drinks/week | 1.00 | 0.98, 1.02 |  | 1.00 | 0.98, 1.02 |  | 1.00 | 0.98, 1.03 |
| Alcoholic binges/week | 1.10 | 0.93, 1.30 |  | 1.09 | 0.92, 1.29 |  | 1.08 | 0.91, 1.29 |
| Depression | 39.90*** | 22.95, 69.40 |  | 37.35*** | 21.41, 65.17 |  | 42.22*** | 24.22, 73.60 |
| Note: *N* = 10,480. **p* < 0.05, ***p* < 0.01, ****p* < 0.001. | | | | | | | | |

| **Supplemental Table 4**. Moderation analysis showing that sleep duration does not significantly moderate the association between insomnia and frequency of suicidal ideation in the combined sample (NHANES 2005-2006 & 2007-2008 cycles). | | | |
| --- | --- | --- | --- |
|  | *OR* | 95*% CI* | *p*-value |
| **Model 1** |  |  |  |
| Insomnia | 1.14* | 1.01, 1.29 | 0.029 |
| Sleep duration (hours) |  |  |  |
| <= 4 | 4.26** | 1.82, 9.97 | 0.002 |
| 5 | 1.42 | 0.57, 3.53 | 0.433 |
| 6 | 1.23 | 0.63, 2.40 | 0.527 |
| 7 | --- |  |  |
| 8 | 1.58 | 0.74, 3.36 | 0.224 |
| 9 | 1.94 | 0.67, 5.67 | 0.213 |
| >= 10 | 1.22 | 0.33, 4.56 | 0.762 |
| Sleep duration X insomnia |  |  |  |
| <= 4 | 0.87 | 0.74, 1.02 | 0.089 |
| 5 | 0.97 | 0.82, 1.15 | 0.712 |
| 6 | 0.98 | 0.85, 1.12 | 0.718 |
| 7 | --- |  |  |
| 8 | 0.98 | 0.84, 1.13 | 0.725 |
| 9 | 0.98 | 0.75, 1.29 | 0.890 |
| >= 10 | 1.13 | 0.89, 1.44 | 0.308 |
| **Model 2** |  |  |  |
| Insomnia | 1.14*** | 1.09, 1.19 | 0.000 |
| Depression | 73.56*** | 27.49, 196.80 | 0.000 |
| Depression X insomnia | 0.90 | 0.81, 1.00 | 0.053 |
| Note: Each model includes adjustments for age, gender, race, education, poverty-to-income ratio, marital status, smoking status, alcohol consumption, and binge drinking. Model 1 is adjusted for depression and Model 2 is adjusted for sleep duration. *N* = 10,480. **p* < 0.05, ***p* < 0.01. | | | |
